# Safety profiles of cyclosporine and tacrolimus in pregnancy: a pharmacovigilance analysis using the FDA Adverse Event Reporting System

**DOI:** 10.3389/fmed.2026.1917080

**Published:** 2026-09-03

**Authors:** Yanhong Deng, Senling Feng, Shengying Shi, Zhongwen Yuan, Zhengrong Mei, Jinjin Yin, Dongdong Jing, Shaozhi Liu

**Affiliations:** Department of Pharmacy, Guangdong Provincial Key Laboratory of Major Obstetric Diseases, Guangdong Provincial Clinical Research Center for Obstetrics and Gynecology, The Third Affiliated Hospital, Guangzhou Medical University, Guangzhou, China

**Keywords:** cyclosporine, disproportionality analysis, pharmacovigilance, pregnancy, safety profiles, tacrolimus

## Abstract

**Background:**

The increasing use of calcineurin inhibitors (CNIs) among pregnant women highlights the importance of continuous safety surveillance. This study aimed to characterize the real-world safety signals of cyclosporine (CsA) and tacrolimus (TAC) during pregnancy using data from the FDA Adverse Event Reporting System (FAERS) database.

**Methods:**

We applied four disproportionality data-mining algorithms (reporting odds ratio, proportional reporting ratio, Bayesian confidence propagation neural network, and empirical Bayesian geometric mean) and performed sensitivity analyses restricted to monotherapy cases to minimize confounding by concomitant medications.

**Results:**

A total of 761 CsA-associated and 2,204 TAC-associated pregnancy cases were identified in the FAERS database. Disproportionality analyses yielded 80 positive signals for CsA and 116 for TAC. Both drugs shared signals for common AEs, such as low birth weight, preterm birth, fetal growth restriction, gestational hypertension, and preeclampsia, as well as signals consistent with known nephrotoxicity and neurotoxicity. Comparative signal detection showed that CsA signals were predominantly associated with kidney transplant rejection and elevated creatinine, whereas TAC signals were predominantly associated with thrombotic microangiopathy.

**Conclusion:**

This pharmacovigilance study demonstrates that CsA and TAC share overlapping AE signal profiles during pregnancy, especially for maternal/fetal complications, nephrotoxicity, and neurotoxicity, yet they also exhibit divergent signals that may reflect differences in their safety profiles. Given the exploratory nature of disproportionality analysis, confirmation through prospective clinical studies is warranted.

## 1 Introduction

Calcineurin inhibitors (CNIs), including cyclosporine (CsA) and tacrolimus (TAC), constitute the cornerstone of immunosuppressive therapy in solid organ transplantation and for immune-mediated diseases. By inhibiting calcineurin and blocking T-cell activation, these agents control disease activity and prevent graft rejection (1). However, CNIs are also associated with a range of overlapping adverse effects, including nephrotoxicity, neurotoxicity, impaired glucose metabolism, hypertension, and infection (2–4).

Given the widespread and often prolonged use of CNIs in organ transplantation and rheumatic diseases, their safety profiles during pregnancy have attracted considerable clinical attention. Although both CsA and TAC cross the placental barrier, the precise extent of transplacental transfer remains poorly characterized, raising concerns about potential fetal risks. The U.S. Food and Drug Administration categorized CNIs as Pregnancy Category C (indicating that human risk cannot be ruled out) owing to the potential for increased fetal complications, including preterm birth, low birth weight (LBW), congenital anomalies, and fetal distress (5, 6). Nevertheless, no definitive studies have confirmed teratogenicity attributable to either CsA or TAC in humans, and rates of major fetal malformations appear comparable to those in the general population (7, 8). Moreover, several observational studies and meta-analyses have suggested that CNIs may be relatively safe during pregnancy (9–11), prompting clinical guidelines in both organ transplantation and rheumatology to recommend the continuation of CNI therapy throughout gestation (12–14).

Despite their shared mechanism of action and recommended use in pregnancy, accumulating evidence suggests that CsA and TAC may differ in their safety profiles. Small studies have reported increased rates of hypertensive complications among CsA-treated patients during pregnancy, which can lead to adverse outcomes such as fetal growth restriction (15, 16). Nulman et al. (17) reported that neonates exposed to CsA in utero had higher risks of LBW and preterm birth compared with unexposed controls. Similarly, TAC exposure has been linked to elevated risks of preeclampsia and renal impairment (9), as well as an increased incidence of neonatal hyperkalemia and kidney injury following in utero exposure (7). Furthermore, TAC appears to be more diabetogenic than CsA (18, 19). However, comparative studies of the two agents have yielded conflicting results. A subgroup analysis by Valentin et al. (5) found comparable rates of live births, preterm birth, and miscarriage between the CsA and TAC groups. A 2021 meta-analysis offered more nuanced findings, demonstrating that TAC was associated with a reduced risk of gestational hypertension, whereas CsA was associated with lower cesarean delivery rates and improved live birth outcomes; nonetheless, both agents carried equivalent risks of preeclampsia, gestational diabetes, preterm birth, and LBW (20). Most recently, a comprehensive 2022 systematic review and meta-analysis of 4,450 pregnant patients confirmed that CNI exposure increases the risks of preterm birth and LBW, and identified CsA use, maternal hypertension, and advanced maternal age as independent risk factors for preeclampsia (21).

With the increasing clinical use of CNIs during pregnancy, comprehensive real-world safety evaluations are urgently needed. Several pharmacovigilance studies utilizing the FDA Adverse Event Reporting System (FAERS) database have investigated adverse events (AEs) associated with CsA and TAC (22–24). However, these analyses primarily focused on specific system organ classes in general populations or non-pregnant organ transplant recipients. The pregnancy subpopulation, which exhibits distinct physiological and pharmacokinetic alterations, has remained largely unexplored. To address this gap, we systematically evaluated the comparative safety profiles of CsA and TAC in pregnant women using four complementary disproportionality algorithms (ROR, PRR, BCPNN, and EBGM). This pharmacovigilance study sought to contribute real-world evidence to the ongoing evaluation of CNI safety during pregnancy.

## 2 Methods

### 2.1 Data sources and preprocessing

**Figure 1:**
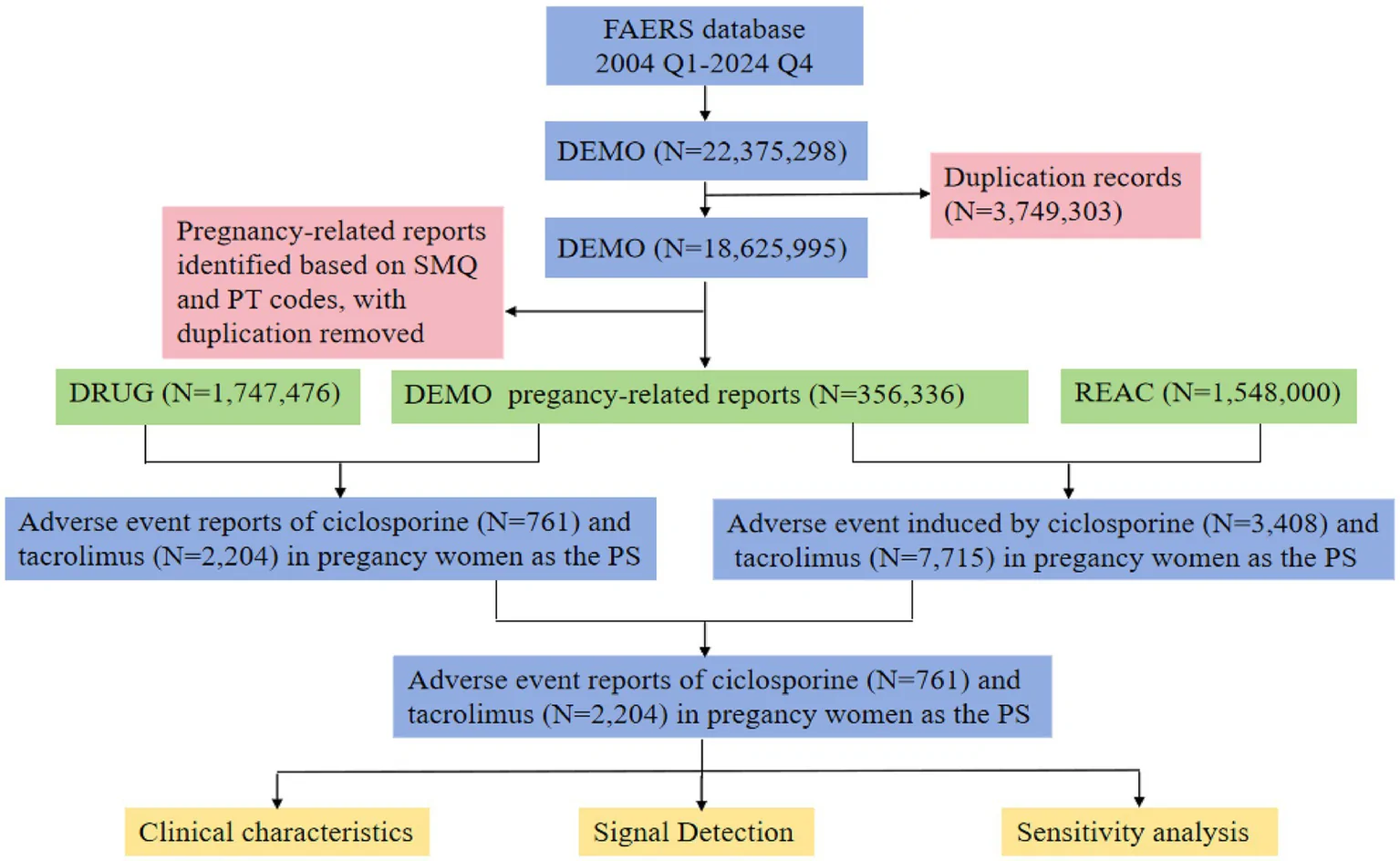
Flowchart of data processing in this study.

The data for this study were obtained from the publicly accessible FAERS database, covering the period from Q1 2004 to Q4 2024. The database comprises seven structured files: patient demographics (DEMO), medication records (DRUG), adverse events (REAC), clinical patient outcomes (OUTC), report sources (RPSR), drug therapy timelines (THER), and therapeutic indications (INDI). We first applied the FDA-recommended data deduplication protocol: for reports sharing the same CASEIDs, only the record with the most recent FDA_DT was retained; when both CASEID and FDA_DT were identical, the report with the highest PRIMARYID was selected as the definitive record (25). We then followed a previously established methodology to identify reports pertaining to pregnant women within the FAERS database (26–28). The screening process for pregnancy-related AEs is illustrated in Figure 1; reports listing CsA or TAC as the “primary suspect (PS)” drug were included. All AEs in the FAERS database were coded and categorized using Preferred Terms (PTs) and System Organ Class (SOC) as defined by the Medical Dictionary for Regulatory Activities (MedDRA) version 27. Data analysis was performed using R software version 4.3.2.

### 2.2 Statistical analysis

Signal detection was performed using disproportionality analyses based on a standard two-by-two contingency table (Supplementary Table S1). To ensure robust signal identification, we implemented a multi-method consensus strategy incorporating the reporting odds ratio (ROR), the proportional reporting ratio (PRR), the Bayesian confidence propagation neural network (BCPNN), and the empirical Bayesian geometric mean (EBGM) (22). A signal was considered confirmed only when it concurrently satisfied the significance criteria for all four algorithms: (1) (ROR025) > 1 with *N* ≥ 3; (2) PRR ≥ 2, *χ*^2^ ≥ 4, and *N* ≥ 3; (3) (IC025) ≥ 0; and (4) (EBGM05) ≥ 2 (29, 30). Requiring concordant positivity across all four algorithms reduces false-positive signals by combining outlier-sensitive frequentist methods with shrinkage-stabilized Bayesian estimates, thereby yielding a more conservative and reliable signal set than any single algorithm alone. The formulas for these four detection methods and their positivity criteria are detailed in Supplementary Table S2. For the BCPNN method, signal strength was graded as follows: weak if 0 < IC025 ≤ 1.5; moderate if 1.5 < IC025 ≤ 3.0; and strong if IC025 > 3.0 (24).

To investigate differences in AE signals between CsA and TAC, we utilized the ROR method in combination with the chi-square test (31, 32). We retained PTs identified as positive signals in CsA-related AE reports and compared them with the corresponding signals for TAC. The ROR in this pharmacovigilance setting differs slightly from its conventional epidemiological application, and the two-by-two table for this comparative ROR algorithm is presented in Supplementary Table S3. The thresholds for detecting significant differences in AE signals between the two drugs using ROR values and chi-square tests are detailed in Supplementary Table S4. Baseline characteristics of the TAC and CsA groups were compared using the Mann–Whitney *U*-test for continuous variables and Pearson’s *χ*^2^ test or Fisher’s exact test for categorical variables (Fisher’s exact test was applied when more than 20% of expected cell counts were less than 5), as appropriate. A two-sided *p*-value less than 0.05 was considered statistically significant. All data management and statistical analyses were conducted using R software (version 4.3.2) and Microsoft Excel.

### 2.3 Sensitivity analysis

To minimize confounding by concomitant medications and assess the robustness of the primary findings, we conducted a sensitivity analysis restricted to monotherapy cases. Specifically, we included only AE reports in which CsA or TAC was listed as the sole drug, systematically excluding all reports containing any other concomitant or co-suspect medication (regardless of reported role). No further inclusion or exclusion criteria were applied. This approach minimized confounding attributable to drug–drug interactions and cumulative adverse effects. The same analytical methodology and significance thresholds used in the primary analysis were applied to determine whether the safety signals remained consistent under single-drug exposure conditions.

## 3 Results

### 3.1 Descriptive analysis

**Figure 2:**
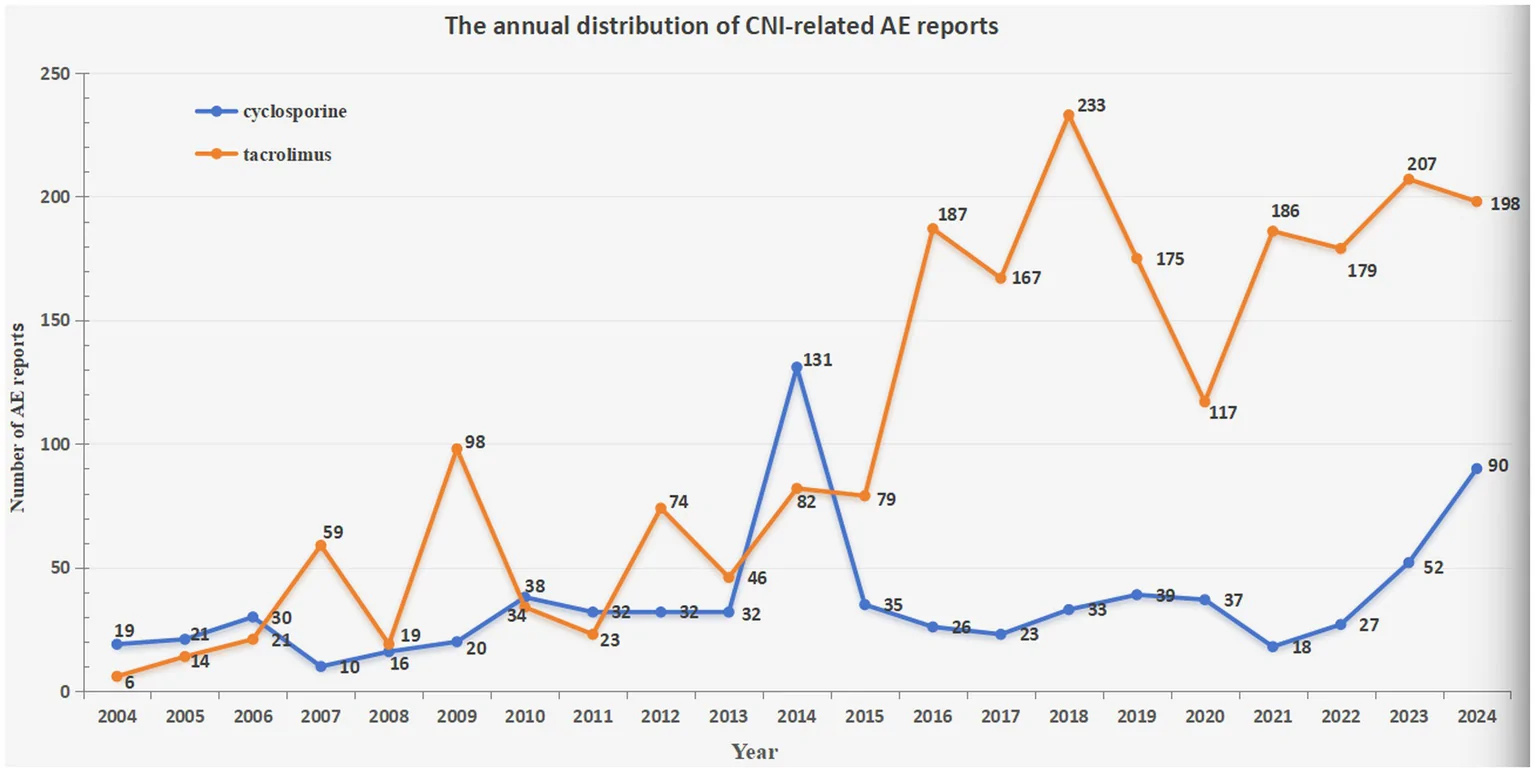
Annual distribution of CNI-related AE reports in pregnant patients from the FAERS database (2004–2024).

A total of 22,375,298 reports were recorded in the FAERS database from 2004 Q1 to 2024 Q4. Following the methods described in previous literature, we identified 356,336 AE reports related to pregnant women, of which 761 cases involved CsA as the PS drug, and 2,204 cases involved TAC as the PS drug. The baseline characteristics of AE reports for both drugs in pregnant patients are presented in Table 1, and the annual reporting time trends are shown in Figure 2. The median patient age for both CsA and TAC was 29 years, with the majority of patients aged 26–35 years; no significant difference was observed between the two groups. Notably, age data were missing for nearly half of the reports (48.36% for CsA and 50.68% for TAC), which warrants caution when interpreting age-related findings. Healthcare professionals submitted a substantial share of reports, although the proportion was significantly higher for TAC than for CsA (83.62% vs. 41.13%), indicating a potentially more reliable reporting profile for TAC. The most frequent reporting regions for both medications were Japan and the United States. The proportion of serious AEs was significantly higher for TAC than for CsA (79.90% vs. 68.46%); among serious AE categories, hospitalization and congenital anomaly rates also differed significantly between the two drugs. The five most frequently reported indications for both drugs were renal transplantation, immunosuppressant drug therapy, immunosuppression, liver transplantation, and systemic lupus erythematosus. The top three concomitant medications for both drugs were corticosteroids, azathioprine, and mycophenolate. As shown in Figure 2, CsA-related AE reports peaked at 131 cases in 2014, declined, and then rose again from 2021 through 2024. TAC exhibited a higher overall report volume, increasing continuously from 2004 to 2018, declining slightly thereafter, and resuming an upward trajectory from 2020 onward.

**Table 1:** Demographic characteristics of AE reports for cyclosporine and tacrolimus in pregnant patients from the FAERS database (Q1 2004–Q4 2024).

Characteristics	Cyclosporine (*N* = 761)	Tacrolimus (*N* = 2,204)	Statistics^b^	*p*-value^d^
Age, *n* (%)				
Mean (SD)	27.30	25.90	219,582^a^	0.349^a^
Median (Q1, Q3)	29 (24, 34)	29 (22, 34)	—	—
0–15	49 (6.44)	204 (9.26)	5.752	0.016
16–25	67 (8.80)	163 (7.40)	1.569	0.210
26–35	198 (26.02)	499 (22.64)	3.589	0.058
36–45	73 (9.59)	179 (8.12)	1.574	0.210
46–55	6 (0.79)	38 (1.72)	3.388	0.066
Missing	368 (48.36)	1,121 (50.86)	—	—
Reporters, *n* (%)				
Consumers	35 (4.60)	302 (13.70)	4.143	0.042
Health professional	313 (41.13)	1843 (83.62)	—	—
Missing	413 (54.27)	59 (2.68)	—	—
Reporter countries (Top 5), *n* (%)				
	Japan, 184 (24.18)	Japan, 486 (22.05)	1.464	0.226
	United States, 74 (9.72)	United States, 413 (18.74)	33.488	<0.001
	United Kingdom, 48 (6.31)	United Kingdom, 153 (6.94)	0.36	0.548
	China, 50 (6.57)	France, 161 (7.30)	—	—
	Germany, 43 (5.65)	Türkiye, 134 (6.08)	—	—
Outcome of AEs, *n* (%)				
Non-serious outcome	240 (31.54)	443 (20.10)	36.927	<0.001
Serious outcome^e^	521 (68.46)	1761 (79.90)	—	—
Life-threatening	9 (1.18)	56 (2.54)	4.867	0.027
Hospitalization	103 (13.53)	435 (19.74)	14.65	<0.001
Disability^c^	1 (0.13)	5 (0.23)	—	1
Death	23 (3.02)	75 (3.4)	0.256	0.613
Congenital anomaly	9 (1.18)	116 (5.26)	23.325	<0.001
Others	376 (49.41)	1,074 (48.73)	0.104	0.747
Indication (Top 5), *n* (%)				
Renal transplant	91 (11.96)	416 (18.87)	19.092	<0.001
Immunosuppressant drug therapy	55 (7.23)	255 (11.57)	11.394	<0.001
Immunosuppression	55 (7.23)	154 (6.99)	0.05	0.824
Liver transplant	31 (4.07)	172 (7.80)	12.343	<0.001
Systemic lupus erythematosus	30 (3.94)	123 (5.58)	3.104	0.078
Concomitant medications (Top3), *n* (%)				
Glucocorticoid	534 (70.17)	1,139 (51.68)	78.674	<0.001
Azathioprine	286 (37.58)	606 (27.5)	27.362	<0.001
Mycophenolate	213 (27.99)	371 (16.83)	44.514	<0.001

Unknown groups are not compared.

a Mann–Whitney *U* test.

b The Pearson chi-square test, chi-squared value (*χ*^2^).

c Fisher’s exact test.

d *p*-value, two-tailed threshold of *p* < 0.05.

e Total serious outcomes may exceed the total number of reported cases because some cases list more than one serious outcome.

### 3.2 Disproportionality analysis

#### 3.2.1 Signal detected at the SOC level

**Figure 3:**
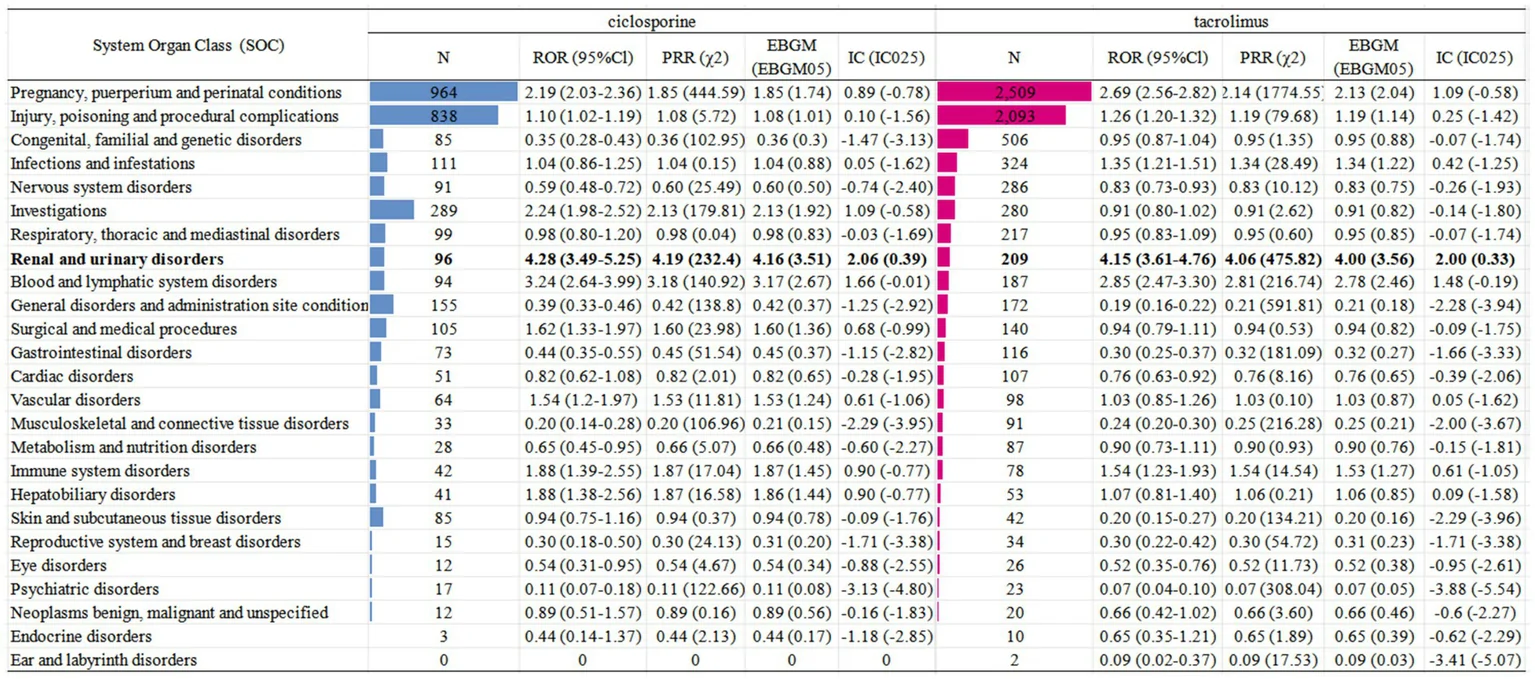
Signal strength of cyclosporine- and tacrolimus-related AEs in pregnant women at the SOC level in the FAERS database (Q1 2004–Q4 2024). Black bold text indicates SOC that meets all four algorithms.

Figure 3 illustrates the signal strengths and report counts for CsA and TAC at the SOC level, revealing similar safety patterns between the two CNIs. For CsA, the most frequently reported SOCs were pregnancy, puerperium and perinatal conditions (*n* = 964, 28.29%), injury, poisoning and procedural complications (*n* = 838, 24.59%), investigations (*n* = 289, 8.48%), general disorders and administration site conditions (*n* = 155, 4.55%), and infections and infestations (*n* = 111, 3.36%). TAC exhibited a comparable distribution, with pregnancy, puerperium and perinatal conditions (*n* = 2,509, 32.52%), injury, poisoning and procedural complications (*n* = 2,093, 27.13%), congenital, familial and genetic disorders (*n* = 506, 6.56%), infections and infestations (*n* = 324, 4.20%), and nervous system disorders (*n* = 286, 3.71%) representing the top five categories. Notably, renal and urinary disorders were the only SOCs that simultaneously met all four disproportionality screening criteria for both drugs, highlighting nephrotoxicity as a consistent and clinically important safety signal shared by CsA and TAC.

#### 3.2.2 Signal detected at the PT level

**Figure 4:**
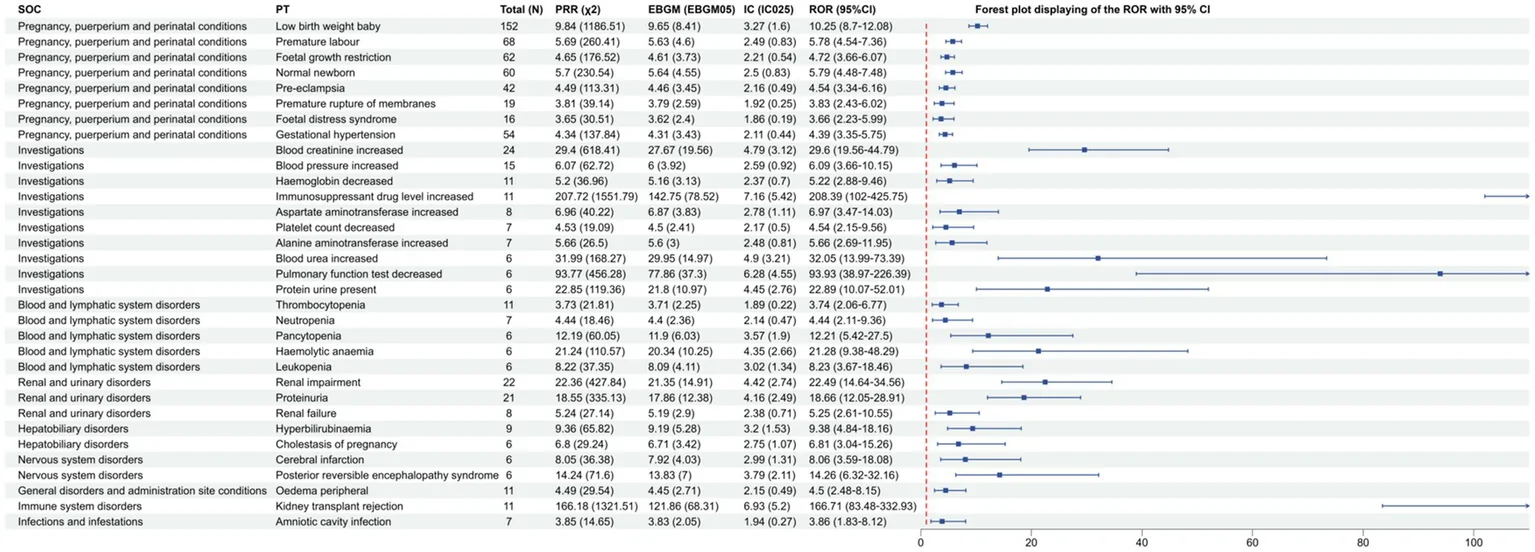
The forest plot of the top 30 signal strengths of CsA-related AEs in pregnant women ranked by case reports at PT levels.

**Figure 5:**
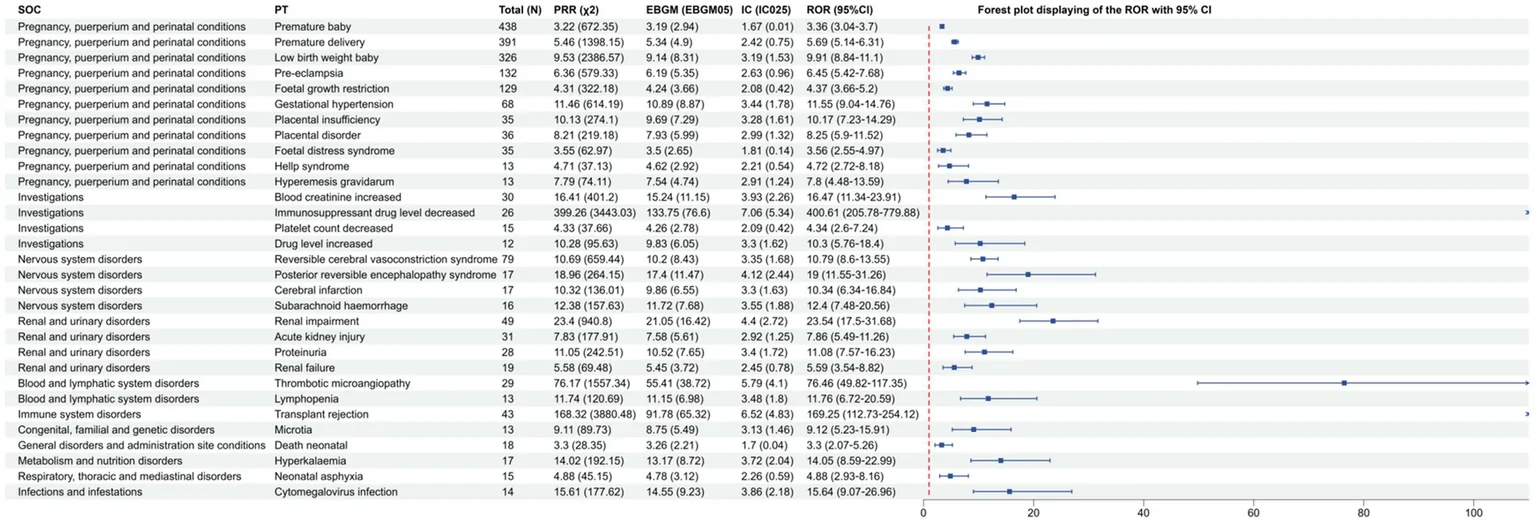
The forest plot of the top 30 signal strengths of TAC-related AEs in pregnant women ranked by case reports at PT levels.

Utilizing four distinct disproportionality algorithms (ROR, PRR, BCPNN, and EBGM), we identified 80 positive signals for CsA and 116 for TAC (Supplementary Tables S5, S6). The reported frequencies and signal strengths for the top 30 most frequently reported AEs for each drug are presented in Tables 2, 3, and the corresponding forest plots are shown in Figures 4, 5.

**Table 2:** The top 30 signal strengths of cyclosporine-related AEs in pregnant women ranked by case reports at PT levels.

SOC/PT	*N*	ROR (95%CI)	PRR (*χ*^2^)	EBGM (EBGM_05_)	IC (IC_025_)
Pregnancy, puerperium and perinatal conditions					
Low birth weight baby	152	10.25 (8.7–12.08)	9.84 (1186.51)	9.65 (8.41)	3.27 (1.60)
Premature labor	68	5.78 (4.54–7.36)	5.69 (260.41)	5.63 (4.60)	2.49 (0.83)
Fetal growth restriction	62	4.72 (3.66–6.07)	4.65 (176.52)	4.61 (3.73)	2.21 (0.54)
Normal newborn	60	5.79 (4.48–7.48)	5.70 (230.54)	5.64 (4.55)	2.50 (0.83)
Gestational hypertension	54	4.39 (3.35–5.75)	4.34 (137.84)	4.31 (3.43)	2.11 (0.44)
Pre-eclampsia	42	4.54 (3.34–6.16)	4.49 (113.31)	4.46 (3.45)	2.16 (0.49)
Premature rupture of membranes	19	3.83 (2.43–6.02)	3.81 (39.14)	3.79 (2.59)	1.92 (0.25)
Fetal distress syndrome	16	3.66 (2.23–5.99)	3.65 (30.51)	3.62 (2.40)	1.86 (0.19)
Investigations					
Blood creatinine increased	24	29.6 (19.56–44.79)	29.40 (618.41)	27.67 (19.56)	4.79 (3.12)
Blood pressure increased	15	6.09 (3.66–10.15)	6.07 (62.72)	6.00 (3.92)	2.59 (0.92)
Hemoglobin decreased	11	5.22 (2.88–9.46)	5.20 (36.96)	5.16 (3.13)	2.37 (0.70)
Immunosuppressant drug level increased	11	208.39 (102–425.75)	207.72 (1551.79)	142.75 (78.52)	7.16 (5.42)
Aspartate aminotransferase increased	8	6.97 (3.47–14.03)	6.96 (40.22)	6.87 (3.83)	2.78 (1.11)
Platelet count decreased	7	4.54 (2.15–9.56)	4.53 (19.09)	4.50 (2.41)	2.17 (0.50)
Alanine aminotransferase increased	7	5.66 (2.69–11.95)	5.66 (26.50)	5.6 (3.00)	2.48 (0.81)
Blood urea increased	6	32.05 (13.99–73.39)	31.99 (168.27)	29.95 (14.97)	4.90 (3.21)
Pulmonary function test decreased	6	93.93 (38.97–226.39)	93.77 (456.28)	77.86 (37.30)	6.28 (4.55)
Protein in urine present	6	22.89 (10.07–52.01)	22.85 (119.36)	21.80 (10.97)	4.45 (2.76)
Blood and lymphatic system disorders					
Thrombocytopenia	11	3.74 (2.06–6.77)	3.73 (21.81)	3.71 (2.25)	1.89 (0.22)
Neutropenia	7	4.44 (2.11–9.36)	4.44 (18.46)	4.40 (2.36)	2.14 (0.47)
Pancytopenia	6	12.21 (5.42–27.5)	12.19 (60.05)	11.9 (6.03)	3.57 (1.90)
Hemolytic anemia	6	21.28 (9.38–48.29)	21.24 (110.57)	20.34 (10.25)	4.35 (2.66)
Leukopenia	6	8.23 (3.67–18.46)	8.22 (37.35)	8.09 (4.11)	3.02 (1.34)
Renal and urinary disorders					
Renal impairment	22	22.49 (14.64–34.56)	22.36 (427.84)	21.35 (14.91)	4.42 (2.74)
Proteinuria	21	18.66 (12.05–28.91)	18.55 (335.13)	17.86 (12.38)	4.16 (2.49)
Renal failure	8	5.25 (2.61–10.55)	5.24 (27.14)	5.19 (2.90)	2.38 (0.71)
Hepatobiliary disorders					
Hyperbilirubinemia	9	9.38 (4.84–18.16)	9.36 (65.82)	9.19 (5.28)	3.20 (1.53)
Cholestasis of pregnancy	6	6.81 (3.04–15.26)	6.80 (29.24)	6.71 (3.42)	2.75 (1.07)
Nervous system disorders					
Cerebral infarction	6	8.06 (3.59–18.08)	8.05 (36.38)	7.92 (4.03)	2.99 (1.31)
Posterior reversible encephalopathy syndrome	6	14.26 (6.32–32.16)	14.24 (71.60)	13.83 (7.00)	3.79 (2.11)
General disorders and administration site conditions					
Oedema peripheral	11	4.50 (2.48–8.15)	4.49 (29.54)	4.45 (2.71)	2.15 (0.49)
Immune system disorders					
Kidney transplant rejection	11	166.72 (83.48–332.95)	166.18 (1321.55)	121.87 (68.32)	6.93 (5.20)
Infections and infestations					
Amniotic cavity infection	7	3.86 (1.83–8.12)	3.85 (14.65)	3.83 (2.05)	1.94 (0.27)

**Table 3:** The top 30 signal strengths of tacrolimus-related AEs in pregnant women ranked by case reports at PT levels.

SOC/PT	*N*	ROR (95%CI)	PRR (*χ*^2^)	EBGM (EBGM_05_)	IC (IC_025_)
Pregnancy, puerperium and perinatal conditions					
Premature baby	438	3.36 (3.04–3.70)	3.22 (672.35)	3.19 (2.94)	1.67 (0.01)
Premature delivery	391	5.69 (5.14–6.31)	5.46 (1398.15)	5.34 (4.90)	2.42 (0.75)
Low birth weight baby	326	9.91 (8.84–11.10)	9.53 (2386.57)	9.14 (8.31)	3.19 (1.53)
Preeclampsia	132	6.45 (5.42–7.68)	6.36 (579.33)	6.19 (5.35)	2.63 (0.96)
Fetal growth restriction	129	4.37 (3.66–5.20)	4.31 (322.18)	4.24 (3.66)	2.08 (0.42)
Gestational hypertension	122	4.42 (3.69–5.30)	4.37 (131.25)	4.30 (3.69)	2.10 (0.44)
Placental insufficiency	35	10.17 (7.23–14.29)	10.13 (274.10)	9.69 (7.29)	3.28 (1.61)
Placental disorder	36	8.25 (5.90–11.52)	8.21 (219.18)	7.93 (5.99)	2.99 (1.32)
distress syndrome	35	3.56 (2.55–4.97)	3.55 (62.97)	3.50 (2.65)	1.81 (0.14)
HELLP syndrome	13	4.72 (2.72–8.18)	4.71 (37.13)	4.62 (2.92)	2.21 (0.54)
Hyperemesis gravidarum	13	7.80 (4.48–13.59)	7.79 (74.11)	7.54 (4.74)	2.91 (1.24)
Nervous system disorders					
Reversible cerebral vasoconstriction syndrome	79	10.79 (8.6–13.55)	10.69 (659.44)	10.20 (8.43)	3.35 (1.68)
Posterior reversible encephalopathy syndrome	17	19.00 (11.55–31.26)	18.96 (264.15)	17.40 (11.47)	4.12 (2.44)
Cerebral infarction	17	10.34 (6.34–16.84)	10.32 (136.01)	9.86 (6.55)	3.30 (1.63)
Subarachnoid hemorrhage	16	12.40 (7.48–20.56)	12.38 (157.63)	11.72 (7.68)	3.55 (1.88)
Investigations					
Blood creatinine increased	30	16.47 (11.34–23.91)	16.41 (401.20)	15.24 (11.15)	3.93 (2.26)
Immunosuppressant drug level decreased	26	400.61 (205.78–779.88)	399.26 (3443.03)	133.75 (76.60)	7.06 (5.34)
Platelet count decreased	15	4.34 (2.60–7.24)	4.33 (37.66)	4.26 (2.78)	2.09 (0.42)
Drug level increased	12	10.30 (5.76–18.40)	10.28 (95.63)	9.83 (6.05)	3.30 (1.62)
Renal and urinary disorders					
Renal impairment	49	23.54 (17.50–31.68)	23.4 (940.80)	21.05 (16.42)	4.40 (2.72)
Acute kidney injury	31	7.86 (5.49–11.26)	7.83 (177.91)	7.58 (5.61)	2.92 (1.25)
Proteinuria	28	11.08 (7.57–16.23)	11.05 (242.51)	10.52 (7.65)	3.40 (1.72)
Renal failure	19	5.59 (3.54–8.82)	5.58 (69.48)	5.45 (3.72)	2.45 (0.78)
Blood and lymphatic system disorders					
Thrombotic microangiopathy	29	76.46 (49.82–117.35)	76.17 (1557.34)	55.41 (38.72)	5.79 (4.10)
Lymphopenia	13	11.76 (6.72–20.59)	11.74 (120.69)	11.15 (6.98)	3.48 (1.80)
Immune system disorders					
Transplant rejection	43	169.25 (112.73–254.12)	168.32 (3880.48)	91.78 (65.32)	6.52 (4.83)
General disorders and administration site conditions					
Death neonatal	18	3.30 (2.07–5.26)	3.30 (28.35)	3.26 (2.21)	1.70 (0.04)
Metabolism and nutrition disorders					
Hyperkaliemia	17	14.05 (8.59–22.99)	14.02 (192.15)	13.17 (8.72)	3.72 (2.04)
Respiratory, thoracic, and mediastinal disorders					
Neonatal asphyxia	15	4.88 (2.93–8.16)	4.88 (45.15)	4.78 (3.12)	2.26 (0.59)
Infections and infestations					
Cytomegalovirus infection	14	15.64 (9.07–26.96)	15.61 (177.62)	14.55 (9.23)	3.86 (2.18)
Congenital, familial, and genetic disorders					
Microtia	13	10.53 (6.26–17.70)	10.51 (122.6)	10.03 (6.49)	3.33 (1.65)

For CsA, among the top 30 PTs ranked by report counts, eight were related to pregnancy, puerperium, and perinatal conditions. The most frequently reported AEs were LBW (*n* = 152), premature labor (*n* = 68), and fetal growth restriction (*n* = 62). Ten PTs belonged to the investigations SOC, with blood creatinine increased (*n* = 24), blood pressure increased (*n* = 15), hemoglobin decreased (*n* = 11), immunosuppressant drug level increased (*n* = 11), and aspartate aminotransferase increased (*n* = 8) being the most commonly reported AEs. Based on IC_025_ values, the strongest signal was immunosuppressant drug level increased [IC (IC_025_) = 7.16 (5.42)]. In the blood and lymphatic system disorders, the most frequent AEs were thrombocytopenia (*n* = 11), neutropenia (*n* = 7), and pancytopenia (*n* = 6). Within the renal and urinary disorders system, renal impairment (*n* = 22), proteinuria (*n* = 21), and renal failure (*n* = 8) were the primary reported AEs, among which renal impairment [IC (IC_025_) = 4.42 (2.74)] and proteinuria [IC (IC025) = 4.16 (2.49)] exhibited the strongest signals. Two PTs were classified under the Nervous System Disorders SOC: cerebral infarction (*n* = 6) and posterior reversible encephalopathy syndrome (PRES) (*n* = 6). Notably, cerebral infarction is not listed in the drug’s prescribing information. Additional frequently reported PTs, including peripheral edema (*n* = 11) and hyperbilirubinemia (*n* = 9), are all listed in the official drug label.

Regarding TAC, among the top 30 PTs by number of case reports, 12 were related to pregnancy, puerperium, and perinatal conditions. The five most frequently reported AEs were premature baby (*n* = 438), premature delivery (*n* = 391), LBW baby (*n* = 326), preeclampsia (*n* = 132), and fetal growth restriction (*n* = 129). In the nervous system disorders SOC, four PTs were identified: reversible cerebral vasoconstriction syndrome (RCVS) (*n* = 79), cerebral infarction (*n* = 17), PRES (*n* = 17), and subarachnoid hemorrhage (*n* = 16), all exhibiting moderate signals. In the investigations SOC, blood creatinine increased (*n* = 30), immunosuppressant drug level decreased (*n* = 26), platelet count decreased (*n* = 15), and drug level increased (*n* = 12) were the most frequently reported AEs, and immunosuppressant drug level decreased exhibited the highest signal intensity (IC_025_ = 4.34). Within the renal and urinary disorders system, the most frequently reported AEs were renal impairment (*n* = 49), acute kidney injury (*n* = 31), proteinuria (*n* = 28), and renal failure (*n* = 19), all of which showed strong signals. In terms of blood and lymphatic system disorders, thrombotic microangiopathy (TMA) (*n* = 29) and lymphopenia (*n* = 13) were the most prevalent AEs, and TMA showed strong signals (IC_025_ = 4.10). Other PTs, such as hyperkalemia, cytomegalovirus infection, and microtia, also exhibited moderate signals, whereas transplant rejection presented a strong signal.

Overall, based on IC025 values, the strongest signals for CsA included increased blood creatinine, increased blood urea, decreased pulmonary function test, and kidney transplant rejection. For TAC, the strongest signals were transplant rejection, fetal damage, decreased immunosuppressant drug levels, and TMA.

#### 3.2.3 Comparison of significant safety signals between CsA and TAC

CsA and TAC shared 26 positive signal PTs, with the most frequently reported being low birth weight baby, fetal growth restriction, gestational hypertension, preeclampsia, and increased blood creatinine (Supplementary Table S7). Among these shared signals, kidney transplant rejection and increased blood creatinine were reported significantly more often with CsA, whereas thrombotic microangiopathy was reported significantly more often with TAC.

#### 3.2.4 Positive PTs of TAC in congenital, familial, and genetic disorders SOC

The analysis identified nine positive PTs for TAC in the congenital, familial, and genetic disorders (Table 4) category, whereas CsA showed no signals in this category. Detailed case-level information is provided in Supplementary Table S8. Importantly, individual case review indicated that nearly all of these positive signals were strongly confounded by concomitant exposure to medications with well-established teratogenic potential, rather than being attributable to TAC itself. Specifically, among the 13 microtia cases, 11 involved concomitant MMF use; all 10 persistent truncus arteriosus cases involved concomitant RAS inhibitor use, with five also receiving CTX; all six external auditory canal atresia cases involved MMF; two of three congenital renal cyst cases involved MMF; and one of the three congenital pneumonia cases involved warfarin. In contrast, hypertrophic cardiomyopathy (*n* = 9), congenital toxoplasmosis (*n* = 5), renal hypoplasia (*n* = 5), and combined immunodeficiency (*n* = 5) were reported without a consistent pattern of concomitant teratogenic medications.

**Table 4:** The positive AEs of tacrolimus in congenital, familial, and genetic disorders system.

PT	*N*	ROR (95%CI)	PRR (*χ*^2^)	EBGM (EBGM_05_)	IC (IC_025_)	Combined teratogenic drugs
Microtia	13	10.53 (6.26–17.70)	10.51 (122.60)	10.03 (6.49)	3.33 (1.65)	11 patients on MMF
Truncus arteriosus persistent	10	11.76 (6.21–22.26)	11.74 (92.83)	11.15 (6.53)	3.48 (1.80)	10 patients on RAS inhibitors, 5 patients on CTX
Hypertrophic cardiomyopathy	9	17.63 (8.92–34.87)	17.61 (129.61)	16.27 (9.19)	4.02 (2.34)	—
External auditory canal atresia	6	8.15 (3.60–18.45)	8.15 (36.15)	7.87 (3.97)	2.98 (1.29)	6 patients on MMF
Congenital toxoplasmosis	5	22.19 (8.81–55.93)	22.18 (91.02)	20.06 (9.26)	4.33 (2.61)	—
Renal hypoplasia	5	5.87 (2.41–14.30)	5.87 (19.63)	5.73 (2.72)	2.52 (0.84)	—
Combined immunodeficiency	5	18.16 (7.27–45.38)	18.15 (74.27)	16.72 (7.77)	4.06 (2.36)	—
Congenital pneumonia	3	7.78 (2.45–24.66)	7.78 (17.06)	7.52 (2.87)	2.91 (1.21)	1 patient on warfarin
Congenital renal cyst	3	8.44 (2.66–26.80)	8.44 (18.86)	8.13 (3.09)	3.02 (1.32)	2 patients on MMF

MMF, mycophenolate mofetil; RAS, renin–angiotensin system; CTX, cyclophosphamide.

### 3.3 Sensitivity analysis

To address potential confounding by concomitant medications, we performed a sensitivity analysis restricted to 130 AE reports for CsA monotherapy and 591 AE reports for TAC monotherapy from the FAERS database. For CsA, 13 positive signals were detected (Supplementary Table S9), of which 7 (53.8%) overlapped with the primary analysis. Persistent signals were observed for key AEs such as LBW baby, normal newborn, fetal growth restriction, preeclampsia, renal impairment, fetal distress syndrome, and PRES. For TAC, we detected 38 positive signals (Supplementary Table S10), of which 30 (78.9%) overlapped with the primary analysis. Persistent positive signals were identified for key AEs including premature baby, LBW baby, premature delivery, fetal growth restriction, gestational hypertension, among others. Specifically, the consistency between the sensitivity and primary analyses was reflected in the persistence of the same key signals across clinically relevant categories, including fetal growth-related events (e.g., LBW baby, fetal growth restriction), hypertensive disorders (e.g., preeclampsia and gestational hypertension), and delivery-related outcomes (e.g., premature baby, premature delivery), demonstrating that the primary findings were robust and not solely attributable to concomitant medication exposure.

## 4 Discussion

The increasing use of CNIs during pregnancy necessitates sustained monitoring of maternal and fetal safety, as the current evidence base relies primarily on small retrospective studies. To address this knowledge gap, this study evaluated the safety of CsA and TAC exposure during pregnancy using FAERS data spanning 2004–2024. The descriptive analysis revealed several notable patterns that warrant consideration before interpreting organ-specific signals. TAC-associated reports were nearly three times more frequent than those for CsA (2,204 vs. 761), likely reflecting contemporary prescribing preferences. The substantial disparity in healthcare professional reporting proportions (83.62% for TAC vs. 41.13% for CsA) suggests that AE profiles may be influenced by the quality of information sources. The higher serious AE proportion for TAC (79.90% vs. 68.46%) may reflect differences in indication severity, regimen intensity, or reporting sources, rather than true pharmacological differences. Moreover, missing age data for nearly half of the reports preclude assessing age as a confounder. The pronounced imbalance in reporter qualification further cautions against direct between-drug comparisons of unadjusted AE profiles.

Although the top five reported indications broadly overlapped (renal transplantation, liver transplantation, immunosuppression, and systemic lupus erythematosus), these categories mask substantial clinical heterogeneity. Renal transplant recipients often have preexisting chronic kidney disease and hypertension, which are risk factors for nephrotoxicity and pregnancy complications such as preeclampsia and fetal growth restriction. Liver transplant recipients exhibit distinct metabolic and coagulation profiles, whereas patients with autoimmune diseases present with variable renal involvement and glucocorticoid exposure. Consequently, the possibility that observed between-drug differences in signal patterns are attributable to confounding by indication or baseline clinical heterogeneity, rather than intrinsic pharmacological differences, cannot be excluded.

Nevertheless, the present study was designed to characterize the safety signal patterns of CNIs in pregnant populations—focusing on maternal/fetal outcomes, congenital anomalies, and organ-specific toxicities—rather than to provide a head-to-head comparative risk assessment in matched cohorts. Stratification by specific indication would have substantially reduced statistical power within the already limited sample sizes, precluding meaningful subgroup analyses. Moreover, the FAERS database lacks granular clinical data on baseline renal function, blood pressure, underlying disease activity, or transplant-specific parameters, preventing adequate adjustment for confounders. Therefore, these findings should be interpreted as exploratory signals; future studies utilizing dedicated pregnancy registries with detailed clinical information are warranted to distinguish drug-specific toxicities from indication-related confounding.

### 4.1 Maternal and fetal outcomes

For both CNIs, the pregnancy, puerperium, and perinatal conditions SOC constituted the largest category of reported AEs. Regarding maternal outcomes, CsA yielded positive signals for premature labor, preeclampsia, premature rupture of membranes, and gestational hypertension, all classified as weak signals, with preeclampsia and gestational hypertension being the most frequently reported maternal complications. In contrast, the detected signals for TAC encompassed premature delivery, preeclampsia, gestational hypertension, placental insufficiency, placental disorder, HELLP syndrome, and hyperemesis gravidarum, among which gestational hypertension [IC (IC_025_) = 3.44 (1.78)] and placental insufficiency [IC (IC_025_) = 3.28 (1.61)] reached moderate signal intensity. For fetal outcomes, the most frequently reported AEs for CsA were LBW, fetal growth restriction, and normal newborn, with LBW [*n* = 152, IC (IC025) = 3.27 (1.60)] reaching moderate signal intensity. Similarly, for TAC, the most frequent AEs were preterm birth, LBW, and fetal growth restriction, with LBW [*n* = 326, IC (IC025) = 3.19 (1.60)] also demonstrating moderate signal intensity.

Transplant recipients are known to have an increased prevalence of hypertension, preeclampsia, preterm birth, and LBW, with preeclampsia rates substantially exceeding those observed in the general population (33, 34). The elevated risk for preterm delivery and LBW can be attributed to the higher incidence of pregnancy-related hypertension and preeclampsia (35). Although underlying disease-related factors contribute to this increased risk, the influence of immunosuppressive medications cannot be excluded (34). Early studies have demonstrated that CNI use during pregnancy in transplant recipients was associated with hypertension (16, 35–37). Accurate diagnosis of preeclampsia in transplant recipients can be challenging because of the confounding effects of preexisting proteinuria and hypertension (33, 37). A systematic review and meta-analysis revealed that CNI-treated patients had an increased risk of preeclampsia compared with the general population, and identified CsA use, maternal hypertension, and advanced maternal age as independent risk factors for preeclampsia (21). A recent study observed higher rates of CNI use (particularly TAC) among women with preeclampsia; however, after adjusting for era, no significant association was found between CNI exposure and preeclampsia (38). This finding may reflect the physiological decline in total CNI levels during pregnancy, which necessitates dose increases that can result in elevated free CNI concentrations, thereby causing nephrotoxicity that may mimic preeclampsia (38, 39). Additionally, both CsA and TAC induce endothelial dysfunction through distinct mechanisms that contribute to pregnancy complications and have been implicated in the pathogenesis of preeclampsia (33, 40). Regarding differences in safety between CsA and TAC during pregnancy, direct comparative studies remain limited. Although our study identified more reports of LBW, fetal growth restriction, and gestational hypertension with CsA, and more reports of preeclampsia with TAC, these differences did not reach statistical significance. These findings partially align with Gong et al. (20), who similarly reported no significant differences in preeclampsia rates but found TAC to be associated with a reduced risk of gestational hypertension—a finding that contrasts with the present study’s findings. Hypertension and preeclampsia during pregnancy pose significant risks for adverse maternal and fetal outcomes, including preterm birth, fetal growth restriction, and allograft loss (41). Clinical management involves initiating antihypertensive therapy for sustained blood pressure ≥140/90 mmHg and considering low-dose aspirin prophylaxis to reduce the risk of preeclampsia (42, 43).

### 4.2 Congenital, familial, and genetic disorders

Although the present pharmacovigilance analysis detected nine positive signals within the congenital, familial, and genetic disorders SOC with TAC and none with CsA, the case-level data presented in Table 4 strongly indicate that nearly all of these signals were attributable to concomitant exposure to medications with well-established teratogenic potential, rather than to TAC itself. This finding reconciles the apparent discrepancy between the observed interdrug difference in signals and the existing clinical evidence that TAC is not a major teratogen (7, 44). Thus, the positive signals for TAC likely reflect prescribing patterns and concomitant medication profiles in the TAC-treated population, rather than a higher intrinsic teratogenic risk compared with CsA.

Detailed case review supported this interpretation. The majority of congenital malformation reports occurred in patients concurrently receiving medications with well-established teratogenic risks, suggesting that these signals were primarily driven by concomitant drug exposure rather than TAC per se. MMF is a confirmed human teratogen known to cause auricular hypoplasia/anotia, external auditory canal atresia, and congenital heart defects (45, 46). In our dataset, 11 of 13 microtia cases, all six external auditory canal atresia cases, and two of three congenital renal cyst cases were on MMF, precisely mirroring this teratogenic spectrum. First-trimester exposure to RAS inhibitors is associated with an increased risk of congenital malformations, particularly cardiovascular and central nervous system defects (47, 48). Characteristic fetal effects include renal failure (oliguria/anuria), oligohydramnios, hypocalvaria, and patent ductus arteriosus (49). Notably, all 10 cases of persistent truncus arteriosus in the present study involved concomitant RAS inhibitor use, with five patients also receiving CTX, suggesting that polypharmacy may significantly increase the risk of complex cardiovascular malformations.

Among the signals, hypertrophic cardiomyopathy stands out as a possible exception warranting further attention. Nine cases were reported without a consistent pattern of concomitant teratogenic drugs. Published case reports describe TAC-associated hypertrophic cardiomyopathy that improved after discontinuation or conversion to CsA or sirolimus (50, 51). A plausible mechanism involves TAC binding to FK-binding proteins, which are present not only in T cells but also in cardiac tissue; calcineurin inhibition in cardiomyocytes may influence sympathetic activation or calcium release channels, potentially contributing to cardiac effects. However, given the exploratory nature of disproportionality analyses and the limited number of cases, these findings should be interpreted as hypothesis-generating rather than as evidence of a causal relationship. Further prospective studies are warranted to clarify whether TAC has a direct effect on cardiac structure and function during pregnancy.

### 4.3 Renal and urinary disorders

The present analysis revealed that the renal and urinary disorders SOC was the only one that simultaneously met all four disproportionality screening criteria for both CNIs. For both CsA and TAC, we detected moderate and strong signals for renal-associated AEs, including increased blood creatinine, renal impairment, proteinuria, and renal failure. These findings align with established mechanisms whereby CNIs cause arteriolar vasoconstriction and endothelial injury, which underlie their well-recognized association with nephrotoxicity and hypertension (2, 52). These findings are consistent with the study by Koenjer et al. (53), which, despite its small sample size, demonstrated an increased risk of elevated serum creatinine levels in pregnant CNI users compared with non-CNI controls. The nephrotoxic effects of CNIs are complex and multifaceted, manifesting through distinct acute and chronic phases. Acute CNI nephrotoxicity occurs primarily through vasoconstrictive mechanisms, characterized by decreased production of vasodilators such as prostaglandin E_2_ and nitric oxide, coupled with increased levels of vasoconstrictors including thromboxane and endothelin, as well as activation of the renin–angiotensin system (2, 54). In contrast, chronic nephrotoxicity involves more complex molecular pathways that operate independently of the NFAT pathway, likely through off-target effects involving the inhibition of serine/threonine protein kinases (55).

Although CsA and TAC share similar mechanisms of action, they may exhibit distinct nephrotoxicity profiles (56). Direct comparison between the two CNIs revealed subtly different patterns of renal toxicity. While no significant differences were observed in reports of renal impairment, proteinuria, or renal failure, increased blood creatinine was significantly more frequent with CsA than TAC [ROR (95% CI) = 1.87 (1.09–3.21), *p* = 0.03]. This finding is consistent with mechanistic studies demonstrating that CsA may cause greater nephrotoxicity than TAC (57, 58). Clinical studies in the general transplant population further support this observation, showing that TAC-treated patients demonstrate superior graft function and better blood pressure control than those receiving CsA (7, 59). Notably, TAC was uniquely associated with acute kidney injury, an AE not observed with CsA that warrants particular attention.

Neonatal nephrotoxicity is an important consideration in evaluating the safety of CNI therapy during pregnancy. In the present study, we found that hyperkalemia [*n* = 17, ROR (95% CI) = 14.05 (8.59–22.99)] was a moderate signal with TAC. Further analysis of the DEMO table revealed that 12 cases occurred in neonates, highlighting the significance of hyperkalemia in this population. TAC has been reported to cause reversible hyperkalemia and renal impairment in neonates (60). Therefore, close monitoring of renal function and electrolytes in exposed newborns is essential. These results carry important clinical implications, as even modest increases in creatinine during pregnancy may indicate significant renal compromise that could adversely affect both maternal and fetal outcomes. Nevertheless, several limitations must be acknowledged. Pharmacovigilance data are inherently subject to reporting bias, and the clinical significance of these observed differences requires validation through prospective studies specifically designed for pregnant populations. Furthermore, the FAERS database does not capture dose–response relationships or temporal associations, both of which are crucial for understanding CNI nephrotoxicity in pregnancy.

### 4.4 Nervous system disorders

This pharmacovigilance analysis revealed that both CsA and TAC were associated with neurological AEs during pregnancy, with TAC exhibiting a broader spectrum and higher frequency of neurological complications. CsA was associated with two primary neurological AE signals: cerebral infarction [*n* = 6, IC (025) = 2.99 (1.31)] and PRES [*n* = 6, IC (025) = 3.79 (2.11)]. TAC exhibited a more extensive neurological safety profile, with four distinct AE signals, all demonstrating moderate signals: RCVS [*n* = 79, IC (025) = 3.35 (1.68)], cerebral infarction [*n* = 17, IC(025) = 4.12 (2.44)], PRES [*n* = 17, IC(025) = 3.30 (1.63)], and subarachnoid hemorrhage [*n* = 16, IC(025) = 3.55 (1.88)]. These results suggest that TAC may be associated with a higher propensity toward neurological complications during pregnancy, with RCVS being the predominant concern. Although both drugs shared signals for cerebral infarction and PRES, TAC was uniquely associated with RCVS and subarachnoid hemorrhage.

These neurological complications, including PRES and cerebral infarction, which are documented in and established by drug labeling, are attributed to the neurotoxic effects of CNIs, although the precise biochemical mechanisms remain poorly understood. Current evidence suggests several potential pathways: CNIs may compromise blood–brain barrier integrity by inducing endothelial apoptosis, stimulating nitric oxide production, and inhibiting P-glycoprotein function, thereby promoting drug accumulation in brain tissue and fluid extravasation into the interstitium; CNI-induced mitochondrial dysfunction may impair cellular energy metabolism, leading to neuronal toxicity; CNI-immunophilin complexes may directly mediate neurotoxic effects; and CNIs may cause vasoconstriction or vascular injury, further contributing to neurological damage (61).

The present analysis identified RCVS as a distinctive positive signal for TAC (*n* = 79), consistent with extensive documentation in the published literature (62–65). Notably, in one case report, substituting CsA for TAC, reducing the methylprednisolone dose, and administering vasospasm-targeted calcium channel blockers (nimodipine and verapamil) successfully resolved symptoms in a patient with TAC-induced RCVS (63). This observation underscores the potential differential vasoconstrictive effects between CNIs in the management of cerebrovascular complications. Prompt neuroimaging with magnetic resonance imaging (MRI) and magnetic resonance angiography (MRA) is essential for patients presenting with severe neurotoxic symptoms, particularly when TAC serum concentrations are elevated. Serial imaging should be considered if symptoms persist or worsen, as early-phase RCVS may escape detection even with MRA (61). Evidence suggests a clear correlation between TAC serum concentrations and neurological symptom severity, supporting immediate drug discontinuation as the preferred strategy for rapid recovery from vasospasm (61). Although the pathophysiological mechanisms of RCVS remain incompletely elucidated, recognized triggers include the puerperium, recreational drugs (e.g., cannabis, cocaine), and medications such as selective serotonin reuptake inhibitors, TAC, and cyclophosphamide (66). TAC-induced vasoconstriction has been proposed as a unifying mechanism underlying its cardiovascular (hypertension), renal, and neurological adverse effects, including headache, PRES, and RCVS (67).

Pregnancy alters TAC pharmacokinetics, complicating therapeutic drug monitoring and potentially increasing the risk of neurotoxicity (68). Moreover, preeclampsia is a common complication in pregnant transplant recipients and can exacerbate neurological complications through endothelial dysfunction (69). From a clinical perspective, these findings suggest that pregnant women receiving TAC require more intensive neurological monitoring than those receiving CsA, with particular attention to early RCVS warning signs. Future studies should focus on elucidating the mechanistic basis of pregnancy-related CNI neurotoxicity and developing evidence-based monitoring and management protocols for this high-risk population.

### 4.5 Blood and lymphatic system disorders

The present analysis identified thrombotic TMA as a significant signal AE associated with both CsA and TAC, with TAC demonstrating a substantially stronger risk profile. TAC was linked to 29 TMA cases compared with only three cases for CsA, and signal detection analysis revealed a strong signal for TAC (IC025 = 4.1) vs. a moderate signal for CsA (IC025 = 2.01). Moreover, comparative analysis revealed a statistically significant difference in the incidence of TMA between CsA and TAC [ROR (95% CI) = 0.24 (0.07–0.79), *p* = 0.018]. Although comparative data on CNI-associated TMA in pregnancy remain scarce (70, 71), these results are consistent with well-documented evidence from the general population (72–74). Notably, Al-Nouri et al. (72) similarly reported higher frequencies of TAC-associated TMA cases compared to CsA in their comprehensive review of drug-induced TMA. Multiple case reports have also documented clinical improvement in patients who developed TAC-induced TMA after switching to alternative immunosuppressants, including CsA or everolimus (73, 74), lending additional support to the differential risk profile between these CNIs.

TMA is a pathological syndrome characterized by the classic triad of microangiopathic hemolytic anemia, thrombocytopenia (platelet count <150 × 10^9^/L), and end-organ damage, with a particular predilection for the renal, cardiac, and nervous systems (75). The underlying pathophysiology involves endothelial injury and subsequent microthrombi formation within small blood vessels, ultimately leading to ischemic organ dysfunction. Drug-induced TMA represents the third most common cause, following pregnancy-related and infection-related forms (76) CNIs are among the most frequently implicated medications due to their direct endothelial toxicity and pro-thrombotic effects. The convergence of pregnancy and CNI therapy creates a particularly high-risk scenario for TMA development. Pregnancy independently predisposes to TMA through multiple mechanisms, including endothelial dysfunction, complement system dysregulation, and the physiologic hypercoagulable state. When CNIs are administered during pregnancy, their inherent thrombotic potential may synergistically amplify the already elevated TMA risk associated with the pregnant state. The consistency between our pregnancy findings and established evidence in the general population reinforces the differential TMA risk profile among CNIs, emphasizing the critical importance of individualized immunosuppressive therapy selection, particularly during pregnancy where both maternal and fetal safety considerations must be carefully balanced against therapeutic efficacy.

### 4.6 Limitations

This study is subject to several inherent limitations characteristic of pharmacovigilance analyses based on FAERS data, and these constraints must be carefully considered when interpreting the present findings. First, FAERS is a spontaneous reporting system that is subject to non-random underreporting, often influenced by event severity, drug novelty, and regulatory or media attention. This can create temporal fluctuations in report counts that are unrelated to actual risk changes. Conversely, milder or chronic events are disproportionately underreported. Critically, the absence of denominator data (i.e., the total number of patients exposed to each drug) makes it impossible to calculate true incidence rates or absolute risks. Consequently, disproportionality metrics (e.g., ROR, PRR) are merely comparative ratios within the reported dataset and should not be interpreted as real-world risk estimates. Second, the completeness and quality of individual reports vary considerably. Many reports lack essential clinical variables, including drug dose, treatment duration, route of administration, gestational age, comorbidities, disease severity, and concomitant medications. These omissions not only limit the ability to adjust for potential confounders but also introduce measurement bias and potential misclassification of both exposures and outcomes. Third, the pregnancy identification approach, based on pregnancy-specific AE terms and documented transplacental drug exposure, is likely to systematically under-identify pregnant women who experience non-specific AEs or whose pregnancy status was not recorded, potentially distorting observed drug–pregnancy associations. The lack of standardized gestational age reporting also prevents trimester-specific analyses that are critical for teratogenicity assessment. Fourth, disproportionality analysis, while useful for hypothesis generation, cannot establish causation. The associations we observed may result from confounding, chance, or reporting artifacts rather than a true causal relationship. Taken together, given these multifaceted limitations, including underreporting, stimulated reporting, missing denominators, incomplete clinical data, confounding by indication, and the non-causal nature of signal detection, the findings should be interpreted as preliminary signals only. Our results are hypothesis-generating and require validation through prospective studies with adequate controls and comprehensive adjustment for confounders.

## 5 Conclusion

This pharmacovigilance study identified 80 and 116 positive signals for pregnancy-related AEs associated with CsA and TAC, respectively. Both drugs exhibited overlapping profiles of frequently reported complications, including LBW, preterm birth, fetal growth restriction, gestational hypertension, and preeclampsia. Safety signals for nephrotoxicity and neurotoxicity were consistent with established drug labeling, with RCVS being the most notable neurological signal for TAC. Comparative analysis revealed divergent safety profiles: CsA was disproportionately associated with kidney transplant rejection and increased blood creatinine, while TAC showed a stronger signal for TMA. These findings characterize the real-world safety profiles of the two CNIs during pregnancy. Given the inherent limitations of spontaneous reporting data, these results are hypothesis-generating and warrant confirmation in prospective clinical studies. Enhanced monitoring of renal function, neurological symptoms, and TMA is recommended for pregnant patients receiving CNI therapy.

## Data Availability

The original contributions presented in the study are included in the article/Supplementary material, further inquiries can be directed to the corresponding authors.

## Glossary

AEs: adverse events
BCPNN: Bayesian confidence propagation neural network
CNIs: calcineurin inhibitors
CsA: cyclosporine
CTX: cyclophosphamide
EBGM: empirical Bayesian geometric mean
FAERS: Food and Drug Administration Adverse Event Reporting System
FDA: Food and Drug Administration
LBW: low birth weight
MedDRA: Medical Dictionary for Regulatory Activities
MMF: mycophenolate mofetil
MRA: magnetic resonance angiography
MRI: magnetic resonance imaging
PRR: proportional reporting ratio
PS: primary suspect
PT: preferred term
RAS: renin-angiotensin system
RCVS: reversible cerebral vasoconstriction syndrome
PRES: posterior reversible encephalopathy syndrome
ROR: reporting odds ratio
SOC: system organ class
TAC: tacrolimus
TMA: thrombotic microangiopathy
